# Prematurity and Low Birth Weight in Neonates as a Risk Factor for Obesity, Hypertension, and Chronic Kidney Disease in Pediatric and Adult Age

**DOI:** 10.3389/fmed.2021.769734

**Published:** 2022-02-03

**Authors:** Maria Agostina Grillo, Gonzalo Mariani, Jorge R. Ferraris

**Affiliations:** ^1^Pediatric Department Hospital Italiano de Buenos Aires, Buenos Aires, Argentina; ^2^Pediatric Nephrology Division, Buenos Aires, Argentina; ^3^Neonatology Division, Buenos Aires, Argentina; ^4^Instituto Universitario Hospital Italiano de Buenos Aires, Buenos Aires, Argentina; ^5^Pediatric Department, Universidad de Buenos Aires, Buenos Aires, Argentina

**Keywords:** preterm neonates, low birth weight, chronic kidney disease, arterial hypertension, fetal programming, extrauterine growth restriction, intrauterine growth restriction, small gestational age

## Abstract

Low weight at birth may be due to intrauterine growth restriction or premature birth. Preterm birth is more common in low- and middle-income countries: 60% of preterm birth occur in sub-Saharan African or South Asian countries. However, in some higher-income countries, preterm birth rates appear to be increasing in relation to a reduction in the lower threshold of fetal viability. The cutoff is at 22–23 weeks, with a birth weight of approximately 500 g, although in developed countries such as Japan, the viability cutoff described is 21–22 weeks. There is evidence of the long-term consequences of prenatal programming of organ function and its relationship among adult diseases, such as hypertension (HT), central obesity, diabetes, metabolic syndrome, and chronic kidney disease (CKD). Premature delivery before the completion of nephrogenesis and intrauterine growth restriction leads to a reduction in the number of nephrons that are larger due to compensatory hyperfiltration and hypertrophy, which predisposes to the development of CKD in adulthood. In these patients, the long-term strategies are early evaluation and therapeutic interventions to decrease the described complications, by screening for HT, microalbuminuria and proteinuria, ultrasound monitoring, and renal function, with the emphasis on preventive measures. This review describes the effects of fetal programming on renal development and the risk of obesity, HT, and CKD in the future in patients with low birth weight (LBW), and the follow-up and therapeutic interventions to reduce these complications.

## Introduction

Preterm birth affects ~11% of births worldwide. The availability of new therapeutics and the increasing complexity of neonatal intensive care units have allowed the survival of infants born at 22 or 23 weeks with birth weights close to 500 g (1–3). The annual prevalence of prematurity in Argentina is between 8 and 9% (4). In this article, we define preterm newborns (PTNs) as those born before 37 week gestational age (GA); small for gestational age (SGA) as neonates with a birth weight less than the 10th percentile for their GA; low birth weight (LBW) and very low birth weight (VLBW) as those with birth weight <2,500 and 1,500 g, respectively, and extremely low birth weight (ELBW) as those with birth weight <1.0 kg.

**Figure 1:**
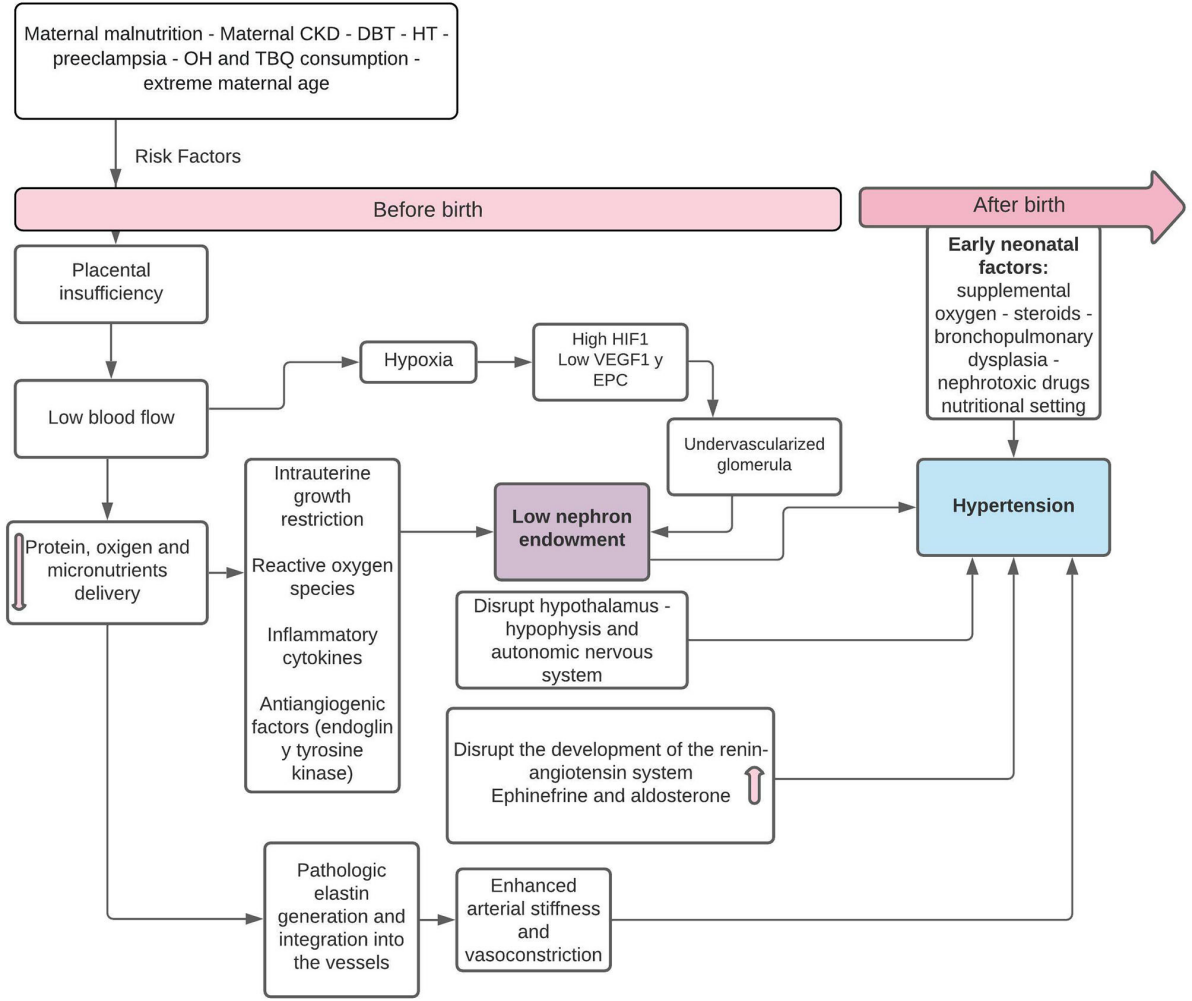
Pathophysiology of fetal “programming” in SGA infants in relation to the decrease in the number of nephrons and postnatal HT. HIF-1, hypoxia-induced factor-1; SGA, small for gestational age; VEGF, vascular endothelial growth factor; EPC, neonatal endothelial progenitor cells. Modified from Stritzke et al. (9) and Crump et al. (11).

Preterm newborns and SGA are particularly vulnerable to the development of hypertension (HT) and chronic kidney disease (CKD). In the former, there is premature exposure to the conditions of extrauterine life, in organs that are not yet prepared for it, where the premature arrest of the development of the vascular tree results in stiffer and narrower arteries, which predisposes to glomerular and endothelial damage, structural alterations due to glomerular hyperfiltration, and increased systolic blood pressure (SBP) in children and adults (5, 6). Preterm infants may also have either an appropriate birth weight for GA or maybe SGA if they experienced superimposed growth restriction. Such growth restriction *per se* is also associated with programming effects in the kidney (7). In SGA infants who have had intrauterine growth restriction (percentile drop throughout pregnancy as a consequence of an alteration in placental circulation), exposure to intrauterine stress generates an altered “fetal programming,” inducing changes at the molecular level and in the functioning of systems, with alterations in renal growth and a decrease in the number of nephrons, which would increase the incidence of HT, CKD, and the risk of metabolic alterations, such as insulin resistance. Pregnancies affected by maternal HT have greater short-term fetal complications, such as fetal death and SGA, as a consequence of placental insufficiency due to preeclampsia (8, 9). The association between preeclampsia and SGA is based on abnormal placental development and decreased placental perfusion, secondary to alteration of the maternal spiral arteries, with spontaneous vasoconstriction of the arteries and placental ischemia, reperfusion-type injury, and oxidative stress (10, 11) (Figure 1).

## Pathophysiology

Nephrogenesis ends at 36 weeks and the reduction in the number of nephrons has consequences for renal health. The number of glomeruli in the normal human embryo increases from week 10, reaches its largest increase between 18 and 32 weeks and is completed between 32 and 36 weeks (12). Each normal kidney has an average of 600–800 thousand glomeruli. Birth weight correlates with the number of glomeruli, estimating an additional 2,32,217 nephrons in each kidney for each 1 kg of birth weight (13, 14). The number of nephrons is reduced by factors that restrict intrauterine growth: micronutrient deficiencies, infections, hypoxia, drugs (nephrotoxic or not, such as beta-lactams), maternal hyperglycemia, glucocorticoids, smoking, or alcohol consumption during pregnancy. The nephron endowment reached at birth will be the one with which the individual will spend the rest of his or her life (13–16).

**Figure 2:**
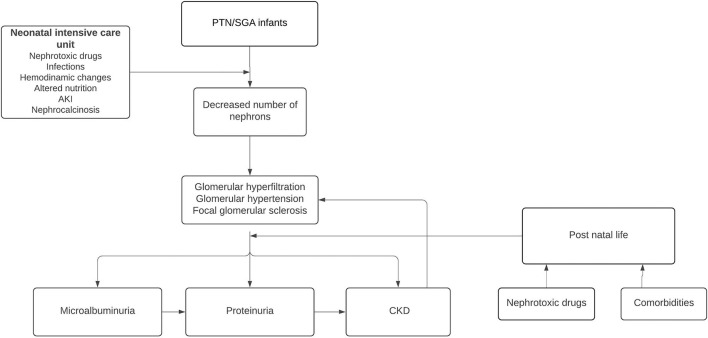
Pathophysiological consequences of altered nephrogenesis in preterm newborns (PTN) and SGA infants.

According to Brenner's hyperfiltration theory (17, 18), humans with a decreased nephron endowment can maintain a normal GFR as individual nephron hypertrophy to increase the total surface area available for renal work. Over time, this adaptive response becomes harmful. The increased glomerular surface area leads to sodium retention and systemic HT and glomerular hyperfiltration disrupts renal autoregulatory mechanisms generating intraglomerular HT (19). These processes render the nephrons sclerotic and this leads to a further decrease in the number of nephrons that reduces the filtration surface, and the remaining nephrons must hypertrophy, manifesting with microalbuminuria and then proteinuria as surrogates of hyperfiltration (12). As a consequence, arterial and glomerular HT is produced, generating glomerulosclerosis, further reducing the number of nephrons (Figure 2). In the terminal phases of CKD, widespread deposition of extracellular matrix in the renal interstitium is recognized as a final common pathway for nephron destruction, resulting from the maladaptive repair of damaged nephrons (20).

Whereas growth restriction increases disease risk in all individuals, often a second hit is required to unmask “programmed” impairments. Programmed disease outcomes are demonstrated more commonly in male offspring compared with females, with these sex-specific outcomes partly attributed to different placenta-regulated growth strategies of the male and female fetus. An extremely common and severe “second hit” for women, known to unmask a variety of conditions in adult life, is pregnancy; it is the greatest physiological “stress test” that a woman can experience in her life. Females who were born small are at an increased risk of pregnancy complications (preeclampsia, gestational diabetes, HT, thyroid, and liver and kidney diseases). The fetus that developed in the womb may also have been exposed to suboptimal conditions and may be programmed to develop the disease in later life, consequences of being born small due to uteroplacental insufficiency. Male fetuses grow at a faster rate than do females and this accelerated growth trajectory makes male fetuses more vulnerable during disturbed pregnancies, with less favorable outcomes occurring throughout the life course of the individual. These sexually dimorphic adaptations are regulated by the placenta. In animal models (e.g., rats), uteroplacental insufficiency results in LBW and programs sex-specific offspring dysfunction and deficits that affect males more than females: development of increased SBP in adult life. This is despite both sexes having decreased nephron number, earlier glomerular hypertrophy, and impaired glucose tolerance, and reduced insulin secretion. Although women are generally less susceptible to programmed disease development, under the physiological demands of pregnancy, various disease states are often unmasked (21).

## Effects of Prematurity on Nephrogenesis

The number of glomeruli is significantly lower in all groups of preterm infants (22). As 60% of nephrons are formed during the third trimester, children born preterm have a significantly lower number of nephrons at birth, which does not catch up adequately postnatally (7). The progression of postnatal nephrogenesis, evaluated in autopsies in preterm infants, evidenced the persistence of glomerulogenesis after birth but altered and with a gradual decrease as postnatal age progressed. Neonates more than 40 days old with acute kidney injury (AKI) showed lower glomerular counts, unlike those with longer survival and without renal failure, but had glomerulomegaly as a compensatory mechanism (23). However, the development of animal models (surgical renal ablation, renal fibrosis, or others) would be important for the study of the effects of prematurity on nephrogenesis (24).

The causes underlying a reduced number of nephrons in an individual are both genetic and environmental. Ongoing interaction between genes and the environment from prenatal to adult life will contribute toward defining the renal potential of an individual. Signaling molecules and transcription factors have been implicated in determining segmental nephron identity and functional differentiation. Whereas some of these genes (p53 gene family, hepatocyte nuclear factor-1) promote the terminal epithelial differentiation fate, others (Notch, Brn-1, IRX, KLF4, and Foxi1) regulate the differentiation of specific nephron segments and cellular types (25). Moreover, epigenetic changes, characterized by alterations in chromatin structure, lead to stable and potentially hereditable changes in gene expression. In particular, DNA methylation has been strongly implicated in fetal renal development and disease (25).

Studies in clinically stable PTN demonstrate that plasma creatinine correlates with GA. Plasma creatinine at birth reflects tubular reabsorption of creatinine. Creatinine increases in the first 36–92 h of life and then gradually decreases. In PTN with GA <32 weeks, the increase in plasma creatinine is greater and the decrease more gradual (being greater in those born with <28 weeks), probably due to a slow progression of glomerular function and tubular creatinine reabsorption (26). Tubular creatinine reabsorption may be a physiological phenomenon in the “immature” kidney due to slow urinary flow and increased creatinine leakage along the immature tubular structures (19).

Preterm newborns may present with AKI events (8–24%) secondary to renal hypoperfusion, asphyxia, respiratory distress syndrome, nephrotoxic drugs exposure (prenatal or postnatal), and infections. In addition, PTNs who are SGA are more vulnerable to renal injury, as SGA has greater nephron depletion and renal dysfunction (22). Decreased glomerular filtration rate (GFR) and increased microalbuminuria have been observed in children and adults who were PTN and SGA, compared with adult PTN but with adequate weight for GA (22).

Epidemiologic studies have shown that incomplete recovery from episodes of AKI constitutes a risk factor for progressive CKD, and CKD, in turn, increases susceptibility to AKI: the proximal tubule, therefore, becomes a primary target of injury and progression of CKD (20). Preterm and critically ill newborns are predisposed to developing AKI because of renal function immaturity and incomplete nephrogenesis in the early postnatal period, which can be irreversibly impaired by drug exposure, and cellular injury to glomeruli or tubules, which may impair repair capacity and increase susceptibility to renal disease later in life (7). In the US, 40% of ICU neonates experienced AKI, it was found that AKI was only recorded in the discharge summary in 13.5% of infants, and none were referred for nephrology follow-up (27). This study illustrated the lack of awareness of the potential long-term impact of neonatal AKI.

On the other hand, PTNs are at risk of extrauterine growth restriction (EUGR), defined as growth below the 10th percentile of growth expectancy, generating consequently greater alterations in nephrogenesis and renal function in adulthood. A lower GFR was evidenced in children examined at 7 years of age (preterm <30 weeks whether SGA or EUGR) compared with children with adequate prenatal and postnatal growth (22). Both intra- and extrauterine growth restrictions were associated with reduced GFR. However, rapid “catchup” growth (i.e., an upward crossing of weight centiles) or increase in BMI leads to the development of higher blood pressure, insulin resistance, and cardiovascular risk already in childhood. These findings are most marked in those who were born small and became relatively larger (28).

Along the same lines, in children aged 1–7 years with a history of PTN and SGA, decreased GFR (78 ± 26.8 ml/min/1.73 m^2^), microalbuminuria (85 ± 187 mg/gr), increased SBP in 21%, and diastolic blood pressure (DBP) in 37% of patients were observed, with mean SBP and DBP between 10 and 15 mmHg above the mean of healthy term newborns. Renal volume increased until 2.5 years of age and then decreased, implying glomerular hypertrophy in the first stage and then possibly glomerular sclerosis (29).

## Long-Term Consequences in the Kidney of the Preterm and SGA Neonate

Early renal complications are related to immaturity in tubular function (tubulopathy of prematurity), presenting inadequate free water management, electrolyte and acid-base imbalance, and mineral and protein losses (30). The increase in GFR that occurs from birth is accompanied by a “parallel” increase in tubular functions to avoid water and solute losses through urine. The activity of the Na+ -K+ -ATPase pump is proportional to GA, which explains the lower reabsorption capacity in PTN <32 weeks (31). Insensible water losses increase in inverse relation to GA. The kidney is in frank natriuresis, inversely proportional to GA, and in PTN <35 weeks, the tubule is unable to conserve sodium. Early onset neonatal hyponatremia in PTNs is secondary to excess water intake associated with increased antidiuretic hormone secretion (30, 31). Serum bicarbonate is lower in PTN (with renal threshold 18 mEq/L) or weight <1,300 g (renal threshold 14 mEq/L); the mechanisms that regulate bicarbonate absorption and secretion have progressive maturation (31).

Late complications with increased risk of CKD, HT, and hypercalciuria in adulthood, are more evident in those PTNs who were born SGA as a consequence of intrauterine growth restriction secondary to placental insufficiency.

## Arterial HT

There is an inverse relationship between birth weight and systolic HT in adolescence (32, 33). A study by Mhanna et al. evaluated blood pressure, obesity, and weight gain as risk factors for HT in 204 patients over 3 years of age, who had been born weighing <1,000 g, with GA of 26 weeks (34). In this population, they found a prevalence of HT of 7.3%, associated with an increase in the body mass index (BMI) and with higher weight gain from birth.

Along the same lines, in another study, over 6,269 PTNs, 528 were SGA and had a higher risk of HT, with the incidence being higher with smaller fetal size. When compared to PTN with adequate birth weight for GA, the SGA had an increased risk of HT of 54% (35).

The risk of presenting HT is also maintained in adulthood. A meta-analysis including preterm-born adults concluded that the mean difference between preterm-born adults and controls was 4.2 mmHg for SBP and 2.6 mmHg for DBP. In another meta-analysis of 1,571 adults born with VLBW (<1,500 g) vs. 777 full-term controls, mean blood pressure averages were higher for subjects <1,500 g; they had 3.4 mmHg higher SBP and 2.1 mmHg higher DBP than controls. The only perinatal event associated with higher blood pressure was maternal preeclampsia (36). These differences are considerable given that, at the population level, it is estimated that a 2 mmHg reduction in SBP results in a 7 to 14% reduction in mortality from ischemic heart disease and a 9 to 19% reduction in mortality from stroke (35–37).

In PTN and SGA patients, a history of breastfeeding was a protective factor for the development of arterial HT; subjects with a birth weight under 2,500 g who were breastfed had a lower prevalence of HT (38). Both breastfeeding during the first months of life and avoiding rapid weight gain in childhood have been shown to prevent the later risk of obesity and dyslipidemia and reduce glucose tolerance (7).

## Chronic Kidney Disease

Reduced nephron endowment and neonatal AKI contribute to the development, HT, and kidney disease (39–42).

Renal function was compared in adolescents born with a history of SGA and mean GA of 27.8 weeks and mean weight of 1,048 g, with adolescents of the same age born at term and birth weight adequate for GA. Whereas there were no differences in blood urea or creatinine values, preterm-born adolescents had a significantly lower GFR compared with term neonates (126.2 vs. 134.3 mL/min/1.73 m^2^). Microalbuminuria was found in 7% of PTN patients, especially in women or in those with a high BMI (40). A meta-analysis, which included more than 2 million individuals, found that a history of SGA was associated with an 80% increased likelihood of microalbuminuria. Another study described a 6.3% increase in the urine albumin–creatinine ratio for every 100 g reduction in birth weight (41, 43).

In adults, the incidence of CKD under 43 years of age, who were born PTN, was evaluated in a large cohort study in Sweden. Of the 4,305 participants, 0.1% had a diagnosis of CKD with the overall incidence rate being 4.95 per 100,000 person-years at all ages examined. The incidences per GA at birth were 9.24 for PTN, 5.90 for early term neonates (37–38 weeks), and 4.47 for full-term neonates (39–41 weeks); PTN and early term neonates had two times the risk of CKD compared with full-term neonates. Moreover, GA was inversely related to CKD risk, with the risk being higher in PTNs and SGA; this association was stronger for the development of CKD in childhood and was maintained in adulthood (44). We can hypothesize that the possibility of CKD will be higher in the extremely preterm neonate (<28 weeks) and very preterm (28–32 weeks) compared with moderate to late preterm (32–37 weeks) since they are born in the period of exponential nephrogenesis and exposed to several risk factors that can compromise its correct development.

A Norwegian birth registry study showed that birth weight less than the 10th percentile for the population was associated with a relative risk of 1.7 for end-stage kidney disease (ESKD) during the first 38 years of life, where LBW was associated with an increased risk of ESKD due to any cause (congenital malformations, hereditary diseases, and glomerular diseases) (45, 46). An investigation in a subgroup aged 18–42 years, excluding subjects with congenital renal disease, found that LBW *per se* was not significantly associated with developing ESKD, but being SGA was. In this Norwegian study among those 18–42 years old, being SGA (birth weight less than 10th percentile for GA) was significantly associated with the risk of ESKD, and the effect was much stronger in those born preterm with SGA than those born at term with SGA (RRs of ESKD of 4.02 and 1.41, respectively). These population level data suggest that both SGA and prematurity are important risk factors and likely potentiate each other's effects, with preterm SGA infants being at the highest risk. (45, 47) On the other hand, renal risk in children born preterm was similar between appropriate GA and SGA and also between VLBW and LBW (25).

## Evaluation, Diagnosis, and Prevention

There are currently no guidelines to identify infants at increased risk of developing CKD due to a low number of nephrons, either congenital or acquired.

However, children and adults who were PTN or SGA need long-term follow-up and early preventive actions to help preserve renal function and CKD. Clinical follow-up should be structured according to greater or lesser risk of developing CKD in the future with the participation of pediatricians and pediatric nephrologists with varying degrees of intervention (48). On the other hand, obstetricians should monitor fetal development, avoiding all risk factors for prematurity and SGA, in close contact with neonatologists (Figure 1).

These interventions should include counseling the parents and then the older patient on how to avoid potentially nephrotoxic drugs exposures (antiinflammatory, antibiotics) (49, 50), other aggravating factors (such as dehydration and urinary tract infection), and control of risk factors for CKD progression (obesity, HT, diabetes, dyslipidemia, anemia, and smoking). HT is an important risk factor for the development of CKD, and effective blood pressure control has been shown to delay the progression of CKD (49). Another risk factor is AKI during the perinatal period with a prevalence between 12.5 and 39.8% in PTN < 1,500 g (51), and with progression to subsequent CKD between 10 and 50% (52).

Early detection of potential indicators of hyperfiltration, such as impaired renal reserve, blunted solute clearance, and microalbuminuria, may provide subtle clues to the presence of reduced nephron number (25).

One follow-up option proposed is as follows in all visits for BP controls, assess growth parameters including BMI, and perform family education on the potential risk of CKD, and continue this follow-up until after adolescence and adulthood (49). BP control should begin before 1 year of age (48) and in children over 5 years of age, control with annual ambulatory blood pressure monitoring should be performed (5, 48).

**Figure 3:**
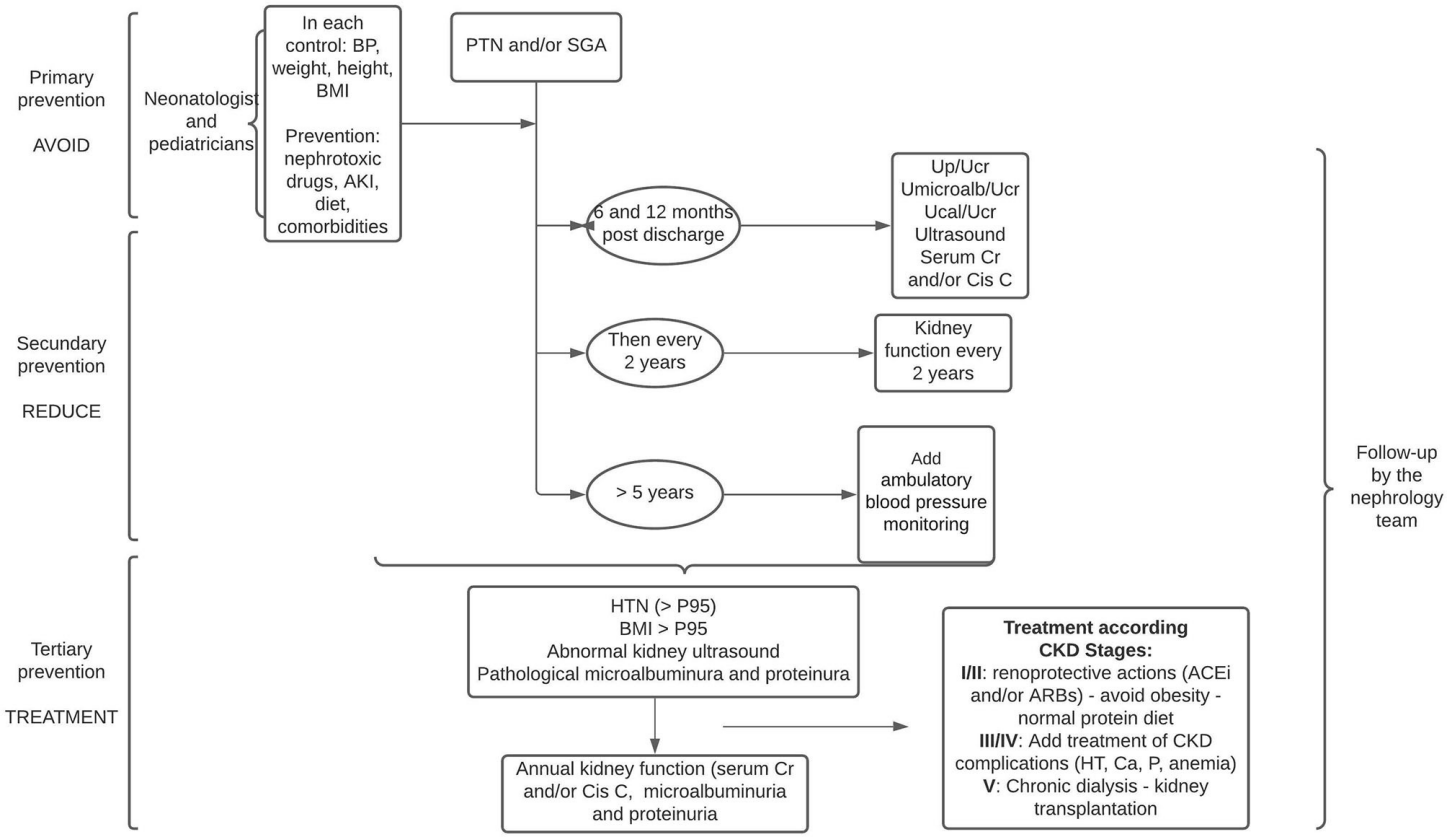
Follow-up proposal for PTN and/or SGA infants in three stages: avoid, reduce, and treat. Cr, creatinine; Cis C, cistatine C; Up/Ucr, urine protein or urine creatinine; UMicroalb/Ucr, urine microalbuminuria or urine creatinine; Ucal/Ucr, urine calcium or urine creatinine; BP, blood pressure; ACEi, angiotensin-converting enzyme inhibitors; ARBs, angiotensin II receptor blockers.

At 6 months after discharge from the neonatal intensive care unit, it is suggested that laboratory tests with serum creatinine and/or cystatin C, and microalbuminuria be performed, and then the periodicity of these tests should be adjusted according to these results or the appearance of comorbidities: history of AKI in the neonatal period or during infancy, HT, obesity, and ultrasound abnormalities. In these cases, blood and urine laboratory controls should be performed annually (48, 49) (Figure 3).

The development of nephrocalcinosis in PTNs confers an additional risk for CKD. Nephrocalcinosis in PTN < 32 weeks and birth weight <1,500 g has a reported prevalence of 7–64% (53), with a resolution of up to 75% within the first year of life (48, 53).

Although some studies describe alterations in renal size on ultrasound monitoring (small kidneys in preterm patients), there is no evidence to indicate systematic ultrasound monitoring. However, baseline ultrasound is recommended to detect small kidneys, renal asymmetries, or structural alterations.

From the age of 18 years, BP, BMI, serum creatinine, and microalbuminuria should be monitored two times a year until the age of 40 years and then annually (7, 48, 49).

Regarding nutritional recovery, rapid growth (catchup) should be avoided to prevent exacerbation of the renal and cardiovascular risk associated with obesity (44, 45). From childhood onward, an adequate “nephroprotective” dietary pattern should be followed, consisting of a reduction in sodium, carbohydrates, saturated fats, and avoidance of excess protein, combined with increased physical activity and restraint of smoking.

Postnatal catchup growth is encouraged in PTNs and SGA in developing countries with the aim of improving resistance to infections, reducing stunting, malnutrition, and reaching normal neurodevelopment. However, this rapid growth can be linear, or present with unbalanced growth in weight and height, with risk of obesity and HT in adulthood (54, 55). Thus, the rapid and continuous upward crossover of weight percentiles during early childhood, with increasing BMI, has been associated with an increased risk of obesity, HT in adulthood, and progression to CKD in PTN, being more accelerated in those who develop obesity (50). HT should be treated aggressively, and in case of microalbuminuria and/or proteinuria, inhibitors of the renin-angiotensin axis should be indicated.

The importance of this very close follow-up will be to implement treatment in the early stages of CKD (1 o 2, cl > 60 ml/min/1.73 m^2^).

The role of strategies played in the clinical management of neonatal intensive therapies in the development of CKD is largely unexplored. Patients are often exposed to medications or situations that compromise nephrogenesis and frequently experience AKI. In recent years, results from the Assessment of Worldwide Acute Kidney Epidemiology in Neonates (AWAKEN) cohort studies have shown the importance of prevention and early detection of AKI given its association with long-term problems (56). If these are independent risk factors for CKD, avoiding nephrotoxins and decreasing the incidence of AKI could lead to better long-term outcomes.

Finally, we must remember that PT and LBW are important risk factors for mortality in childhood and young adulthood (57, 58).

## Conclusion

Hypertension and CKD have a significant impact on overall morbidity and mortality. It is difficult to quantify the impact of fetal programming on these diseases, but both PTN and SGA have been associated with an alteration in nephrogenesis with the consequent decrease in nephrons, so they have a higher risk of CKD in adulthood, with a higher risk at the lower birth weight (up to 70%). We consider that we are facing a “silent epidemic” of CKD in these patients, so preventive strategies should be implemented early to avoid the progression of CKD. This requires not only a multidisciplinary team (obstetricians, neonatologists, pediatricians, nephrologists, neurologists, cardiologists, and nutritionists), but also public and state measures aimed at awareness, information, and prevention. There are gaps that require collaborative, prospective, and randomized research studies in the area, which will help to optimize cost-effective strategies.

## Author Contributions

MG and JF: concept and design and drafting of the manuscript. MG, JF, and GM: acquisitions, analysis, and interpretation of data. GM: critical revision of the manuscript. All authors contributed to the article and approved the submitted version.

## Conflict of Interest

The authors declare that the research was conducted in the absence of any commercial or financial relationships that could be construed as a potential conflict of interest.

## Publisher's Note

All claims expressed in this article are solely those of the authors and do not necessarily represent those of their affiliated organizations, or those of the publisher, the editors and the reviewers. Any product that may be evaluated in this article, or claim that may be made by its manufacturer, is not guaranteed or endorsed by the publisher.
