# Supplementary material for: Prematurity and Low Birth Weight in Neonates as a Risk Factor for Obesity, Hypertension, and Chronic Kidney Disease in Pediatric and Adult Age
Source: Front Med (Lausanne). 2022 Feb 3;8:769734. doi: 10.3389/fmed.2021.769734 (PMC8850406; doi:10.3389/fmed.2021.769734)
Supplement: Supplementary file 1 [file Data_Sheet_1.PDF]

List of acronyms:

TN: term neonates
PTN: preterm neonates.
CKD: chronic kidney disease
IUGR: intrauterine growth restriction
EUGR: extrauterine growth restriction
GA: gestational age
AKI: acute kidney injury
GFR: glomerular filtration rate
SGA: small for gestational age
BMI: body mass index
HT: hypertension
BP: blood pressure
ELBW: extremely low birth weight
